# Neural substrates of cognitive biases during probabilistic inference

**DOI:** 10.1038/ncomms11393

**Published:** 2016-04-26

**Authors:** Alireza Soltani, Peyman Khorsand, Clara Guo, Shiva Farashahi, Janet Liu

**Affiliations:** 1 Department of Psychological and Brain Sciences, Dartmouth College, HB 6207, Dartmouth College, Hanover, New Hampshire 03755, USA

## Abstract

Decision making often requires simultaneously learning about and combining evidence from various sources of information. However, when making inferences from these sources, humans show systematic biases that are often attributed to heuristics or limitations in cognitive processes. Here we use a combination of experimental and modelling approaches to reveal neural substrates of probabilistic inference and corresponding biases. We find systematic deviations from normative accounts of inference when alternative options are not equally rewarding; subjects' choice behaviour is biased towards the more rewarding option, whereas their inferences about individual cues show the opposite bias. Moreover, inference bias about combinations of cues depends on the number of cues. Using a biophysically plausible model, we link these biases to synaptic plasticity mechanisms modulated by reward expectation and attention. We demonstrate that inference relies on direct estimation of posteriors, not on combination of likelihoods and prior. Our work reveals novel mechanisms underlying cognitive biases and contributions of interactions between reward-dependent learning, decision making and attention to high-level reasoning.

## Abstract (web summary)

Humans are often biased in estimating the precise influence of probabilistic events on their decisions. Here, Khorsand and colleagues report a behavioural task that produces these biases in inference and describe a biophysically-plausible model that captures these behavioural deviations from optimal decision making.

## Graphical abstract

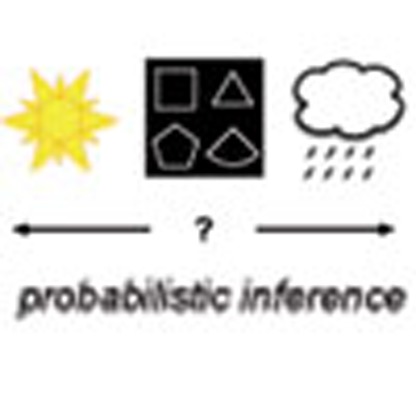

Naturalistic decision making and judgment often require simultaneously learning about and combining evidence from various sources of information. Learning the evidence provided by each source is a non-trivial task when multiple sources are presented at once and feedback is binary (reward versus no reward, success versus failure). For example, examining patients requires a doctor to attend to a variety of symptoms and utilize previous correct/incorrect diagnoses to make proper decisions. Such naturalistic decision making and reasoning situations can be simulated in the laboratory environment using various probabilistic inferences and learning tasks^1^,^2^,^3^. Studies using these tasks have yielded invaluable insights into learning and memory^2^,^4^,^5^,^6^, decision-making processes^7^,^8^,^9^, as well as corresponding neural representations and underlying neural mechanisms^6^,^9^,^10^,^11^,^12^,^13^,^14^.

Interestingly, humans often exhibit systematic biases in their judgment as measured by deviations from normative accounts of inference (for example, computing posteriors by combining evidence and prior information using Bayes theorem). Although these biases have been attributed to heuristics or limitations in cognitive processes^15^, they could reveal neural mechanisms underlying learning, decision making and inference. For example, humans tend to attribute too much predictive power to individual cues preceding the rare outcome when presented with multiple cues simultaneously (a judgment bias known as base-rate neglect^16^). Recently, we proposed a biophysically plausible model that performs probabilistic inference through dopamine (DA)-dependent synaptic plasticity^14^. This model, which was validated by behavioural and electrophysiological data in monkeys^9^, linked base-rate neglect to simultaneous learning of evidence provided by multiple cues. However, it is unclear whether this or other cognitive biases occur in humans due to similar low-level mechanisms, or rather, due to imperfect learning or an inaccurate combination of various sources of information.

Moreover, an important and often neglected aspect of decision making and probabilistic inference is the influence of existing information on what is processed and subsequently updated when reward feedback is received. For example, during a flu epidemic, a doctor may first try to identify a few symptoms that strongly predict a correct diagnosis. For future patients, however, she would not only focus on those symptoms, but also attribute the resulting diagnosis success/failure to those symptoms, rather than the unattended ones. Existing information could influence what should be processed via attentional selection, which in turn could influence future learning and decision processes. Therefore, interactions between reward-dependent learning, decision-making and attentional processes could be crucial for probabilistic inference and could contribute to cognitive biases. Currently, little is known about how these processes interact^17^,^18^, mainly because of the difficulty of separating them^19^; therefore, it is unclear how and to what extent these interactions influence probabilistic inference.

Here we used a combination of experimental and modelling approaches to reveal neural mechanisms underlying probabilistic inference and related biases. First, we used a novel experimental paradigm to precisely measure cognitive biases during both choice and inference. The experiment involved predicting reward on two alternative options based on simultaneously presented cues and subsequently receiving reward feedback. This decision task was followed by an inference task where subjects provided their estimates of the predictive power of individual cues or combinations of cues. Second, we developed a biophysically plausible model of probabilistic inference to simulate subjects' behaviour during our experiment. We find that when alternative options are not equally rewarding, choice behaviour of individual subjects is biased towards the more rewarding option, whereas their inferences about individual cues show the opposite bias. Moreover, inferences about combinations of cues show a bias that depends on the number of cues, contradicting any normative accounts of inference. Our results indicate that inference relies on direct estimation of posteriors and not on combination of likelihoods and prior. We show that reward-dependent learning, decision-making and attentional processes occur concurrently and interact dynamically to determine probabilistic inference and corresponding cognitive biases.

## Results

### Cognitive biases during probabilistic inference

**Figure 1:**
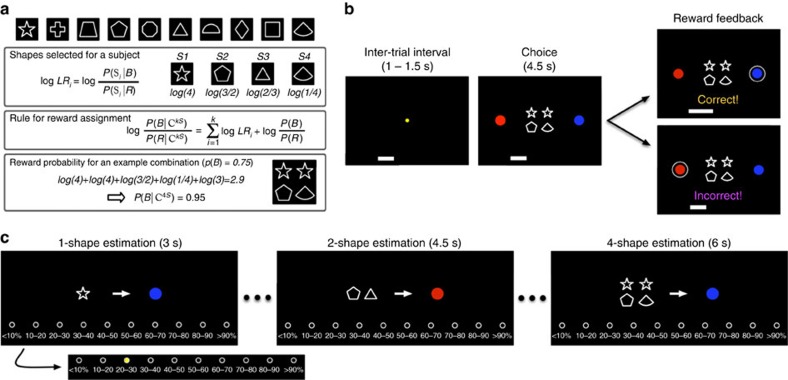
Experimental paradigm and task design. (**a**) The set of all shapes used in the experiment. Insets show an example set of shapes and associated values of logLR for one subject, the rule used to assign reward, an example combination of shapes, and reward probability associated with choosing the blue target for that combination. (**b**) Timeline of a trial during the choice session. The trial starts with the presentation of a fixation point followed by four shapes and two choice alternatives (red and blue targets). A circle appeared around the chosen target followed by the presentation of reward feedback (‘correct' or ‘incorrect') and an update of the reward bar, showing the recent accumulation of reward points. (**c**) Timeline of a trial during the estimation session and the three types of estimates (one-shape, two-shape and four-shape). The subjects used the keyboard to select 1 of 10 values representing the probability that a given shape predicted reward on the red or blue target.

To measure cognitive biases during both choice and inference, we used a novel experimental paradigm in which human subjects simultaneously learned about and combined information from multiple cues through reward feedback, and subsequently provided estimates about the predictive power of individual cues or combinations of cues. Specifically, each subject performed a choice session followed by an estimation session. During each trial of the choice session, the subject was presented with four shapes (selected from a set of 4 shapes with the possibility of repetition) and chose between two alternative options (red or blue targets) to receive reward points (Fig. 1). Subjects learned the information provided by each shape (that is, the probability of collecting reward on the two options) while making decisions about which option is rewarding given a combination of shapes, and subsequently receiving reward feedback. However, for a given subject, either the red or blue target was more often assigned with reward, corresponding to the prior for blue, *P(B)*, equal to 0.25 and 0.75, respectively. During each trial of the estimation session, the subject estimated the probability that the red or blue target would be rewarded given a shape or a combination of shapes (two or four shapes). Importantly, there was no feedback during the estimation session, so subjects relied solely on what they had learned during the choice session when providing estimates.

**Figure 2:**
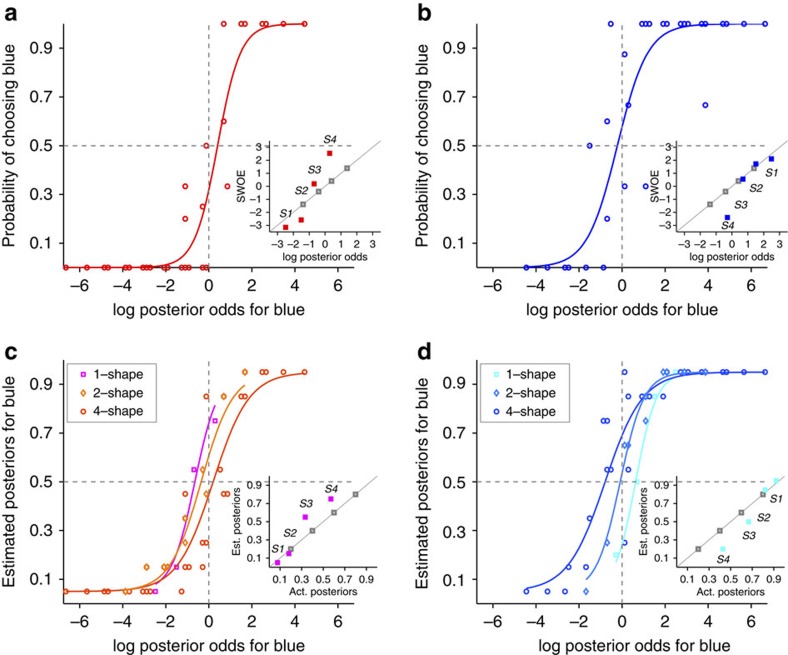
Individual subjects' behaviour during different sessions of the experiment. (**a**,**b**) Choice behaviour of two example subjects for whom the red (**a**) or blue (**b**) target was more rewarding. The psychometric function plots the probability of choosing blue as a function of the actual log posterior odds (for blue) for a given combination of shapes (each symbol represents one such combination). The solid curve shows the fit using a sigmoid function. The inset shows the subjective weight of evidence (SWOE) as a function of the actual log posterior odds for individual shapes, and the diagonal line is shown in solid grey. Both subjects' choice behaviour was biased towards the more rewarding option. (**c**,**d**) Behaviour during the estimation session for the same subjects as in **a** and **b**. The estimation psychometric function (ePF) plots the estimated posteriors as a function of the actual log posterior odds separately for individual shapes or a combination of shapes (the solid curves show the fit using a sigmoid function). The inset shows the estimated posteriors as a function of the actual posterior for individual shapes, and the diagonal line is shown in solid grey. For both subjects, one-shape estimates were biased towards the less rewarding option (note a leftward shift in the ePF for *P(B)*=0.25 and rightward shift for the ePF for *P(B)*=0.75), and the ePFs showed a gradual shift towards the more rewarding option as the number of shapes used for estimation increased.

Even though four shapes were presented on each trial, most subjects performed the task well, as shown by their choice behaviour, and learned the evidence provided by individual shapes (see Methods section for inclusion criteria). Estimation of posteriors for individual shapes matched the order of evidence carried by those shapes in 30 out of 37 subjects, and the remaining 7 subjects missed the order only between 1 pair of shapes. Figure 2a,b shows the behaviour of two example subjects during the choice session. Choice behaviour of each subject was biased towards the more rewarding option in each case: the red target when *P(B)*=0.25 and the blue target when *P(B)*=0.75. We quantified this bias by measuring the indifference point of the psychometric function (PF; see equation 3 in Methods section), *μ*, which was positive for the subject with *P(B)*=0.25 and negative for the subject with *P(B)*=0.75.

However, the same subjects showed an unexpected pattern of biases during the estimation session as revealed by the estimation psychometric function, ePF (Fig. 2c,d). More specifically, even though posterior estimates provided by both subjects were proportional to the log posterior odds, these estimates exhibited systematic biases that depended on the number of shapes used for estimation. In contrast to the bias exhibited during the choice session, one-shape estimates were biased towards the less rewarding option (Fig. 2c,d; note a leftward shift in the ePF for *P(B)*=0.25 and rightward shift for ePF for *P(B)*=0.75). Nevertheless, estimated posteriors were smaller (respectively, larger) than the evidence (shown by grey squares in Fig. 2c,d insets) for *P(B)*=0.25 (respectively, *P(B)*=0.75), indicating that what was learned about individual shapes was influenced by the prior probability but not as strongly as prescribed by Bayes theorem. The subjective weight of evidence (SWOE) for individual shapes, which quantifies the influence of each shape on choice (see Methods section), showed a similar bias towards the less rewarding option (Fig. 2a,b, insets). Finally, the ePF showed a gradual shift towards the more rewarding option as the number of shapes used for estimation increased.

**Figure 3:**
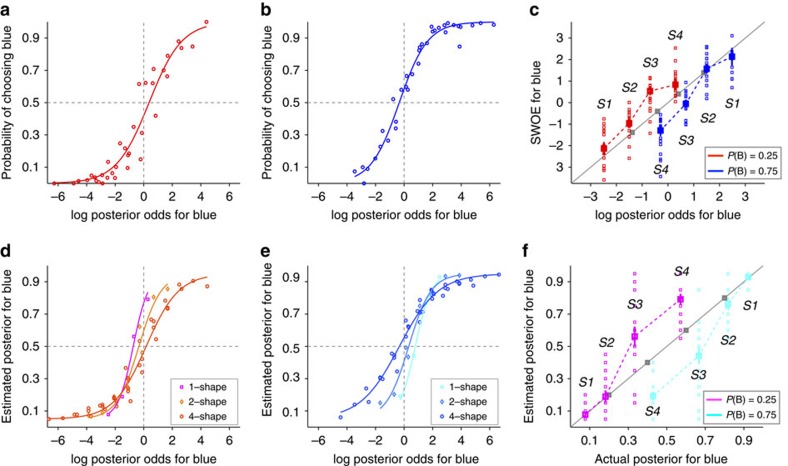
Contradictory biases in choice and estimation. (**a**,**b**) The average psychometric function over all subjects for which the red (**a**) or blue target (**b**) was more rewarding. Conventions are similar to those in Fig. 2. Overall, choice behaviour was biased towards the more rewarding option. (**c**) The SWOEs extracted from individual subjects' behaviour during the choice session are plotted against the actual log posterior odds for each shape (empty symbols). The filled symbols show the average SWOE for each shape across all subjects, and the error bars are the s.e.m. The dashed lines are only to guide the eyes, the grey solid line is the diagonal line, and grey squares show the logLR associated with each shape. Overall, the SWOEs were biased towards the less rewarding option; SWOEs were above the diagonal line for *P(B)*=0.25 and were below the diagonal line for *P(B)*=0.75. (**d**,**e**) The average estimation psychometric function over all subjects for which the red (**d**) or blue target (**e**) was more rewarding. Overall, 1-shape estimates were biased towards the less rewarding option. However, the estimation bias shifted towards the more rewarding option as the number of shapes used for estimation increased. (**f**) Estimated posteriors for individual shapes as a function of the actual posteriors, provided by each subject. The filled symbols show the average posteriors for each shape across all subjects, and the error bars are the s.e.m. The dashed lines are only to guide the eyes, grey solid line is the diagonal line, and grey squares show the evidence associated with each shape.

**Figure 4:**
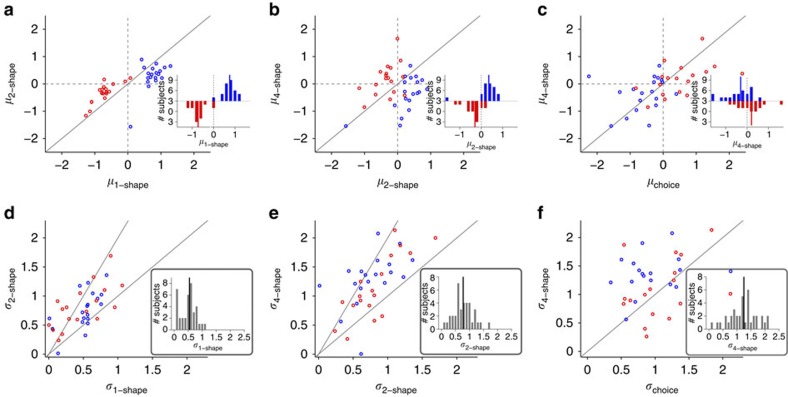
Within-subject changes in the bias and sensitivity to evidence as a function of the number of shapes. (**a**) Plotted are the biases for two-shape estimates (*μ*_2-shape_) versus the biases for 1-shape estimates (*μ*_1-shape_) for individual subjects. Red (respectively, blue) symbols correspond to the bias for subjects for whom the red target (respectively, blue) was more rewarding. The histogram plots the frequency of subjects with a given bias for one-shape estimates, and the solid lines are the median for each set of subjects. (**b**) The same as in **a** but for the bias for four-shape estimates (*μ*_4-shape_) versus the bias for two-shape estimates (*μ*_2-shape_). (**c**) The same as in **a** but for the bias during four-shape estimates (*μ*_4-shape_) versus the bias during the choice session (*μ*_choice_) (**d–f**) Comparison of the stochasticity in choice and estimations, measured by *σ* from the ePF or PF for individual subjects. The lower and higher solid lines show the lines with slope 1 and 2, respectively. The insets show the histogram of *σ* values for one-shape, two-shape and four-shape estimates, and the solid lines show the median for a given distribution.

The aforementioned patterns of systematic biases were observed across all subjects as revealed by the average data across all subjects (Fig. 3) or the distribution of individual subjects' biases (Fig. 4). First, subjects showed contradictory biases during choice and one-shape estimation. More specifically, there was a significant bias in choice towards the more rewarding option (*m*=−0.54±0.70; two-sided sign test *P*=4 × 10^−5^*, N*=37; Figs 3a,b and 4c) and this bias was present for 31 out of 37 subjects (*μ*_choice_=0.50±0.78 for *P(B)*=0.25; *μ*_choice_=−0.58±0.64 for *P(B)*=0.75). At the same time, one-shape estimates were biased towards the less rewarding option (*m*=0.74±0.30; two-sided sign test *P*=6 × 10^−10^, *N*=37; Figs 3d-f and 4a) for all subjects except one (*μ*_1-shape_=−0.72±0.34 for *P(B)*=0.25; *μ*_1-shape_=0.76±0.27 for *P(B)*=0.75). Similar to example subjects (Fig. 2), most subjects estimated posteriors for individual shapes that were smaller (respectively, larger) than the evidence when the red (respectively, blue) target was more rewarding, indicating that what was learned about each shape was influenced by the prior probability but not as strongly as it should be optimally (Fig. 3f). Therefore, although subjects biased their choice towards the more rewarding option, their estimates for individual shapes were biased towards the less rewarding option.

Second, there was a progression of estimation biases towards the more rewarding option as the number of shapes used for estimation increased. The biases for one-shape estimates towards the less rewarding option were larger than those for two-shape estimates (two-sided sign test *P*=10^−8^, *N*=37; Figs 3d,e and 4a) and this was true for all but two subjects (*μ*_2-shape_=−0.33±0.37 for *P(B)*=0.25; *μ*_2-shape_=0.29±0.51 for *P(B)*=0.75). Moreover, the biases for two-shape estimates towards the less rewarding option were larger than those for four-shape estimates (two-sided sign test *P*=4 × 10^−5^, *N*=37; Figs 3d,e and 4b) and this was true for all but five subjects (*μ*_4-shape_=0.16±0.59 for *P(B)*=0.25; *μ*_4-shape_=−0.37±0.63 for *P(B)*=0.75). Interestingly, four-shape estimates were biased towards the more rewarding option (*m*=−0.27±0.61; two-sided sign rank test *P*=0.02, *N*=37), and this bias was slightly less than the bias in choice behaviour; however, the difference between those biases was not statistically significant (two-sided rank-sum test *P*=0.09, *N*=37; Fig. 4c). Therefore, subjects' estimates exhibited systematic biases that depended on the number of shapes used for estimation.

We further examined one-shape estimates corresponding to the extent that individual shapes predicted the reward on a given option, which revealed an important aspect of our data (Fig. 3f). Overall, subjects overestimated the posteriors towards the less rewarding (that is, rare) option, the blue target for *P(B)*=0.25 and the red target for *P(B)*=0.75 (*m*=0.12 across all shapes, two-sided sign test *P*=8 × 10^−4^, *N*=148). This phenomenon has been reported before^1^,^16^,^19^ and is usually referred to as base-rate neglect (that is, a cue that is equally predictive of each outcome is perceived to be more predictive of the less probable outcome) because it is attributed to neglecting the prior probability (that is, base rate) when computing posteriors.

**Figure 5:**
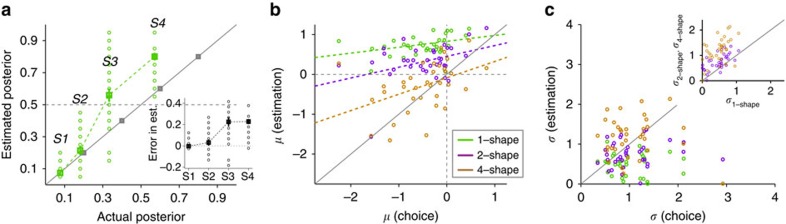
Summary of experimental results. (**a**) Plotted are 1-shape estimates towards the less rewarding option as a function of the actual posteriors, for individual subjects (both *P(B)*=0.25 and *P(B)*=0.75). The inset shows the difference between the estimated and actual posteriors (error in estimation), showing a bias towards the less rewarding option. The large squares show the average over all subjects, and the error bars are the s.e.m. The grey solid line is the diagonal line and the dashed lines are only to guide the eyes. (**b**) Individual subjects' biases during estimation (relative to the more rewarding option) as a function of the bias in choice (relative to the more rewarding option). The colour dashed lines show the linear regression line for the bias in one-shape, two-shape and four-shape estimates as a function of the bias in choice. The grey solid line is the diagonal line. The reductions in the bias baseline from one-shape to two-shape to four-shape estimates show a progression of biases towards the more rewarding option. Correlations between the biases during estimation and choice show that subjects relied on the same information for choice and estimation. (**c**) The relationship between the stochasticity in individual subjects' behaviour during estimation and choice. The inset shows the relationship between *σ* for two-shape and one-shape estimates as well as *σ* for four-shape and one-shape estimates.

Our data revealed two novel aspects of base-rate neglect, in addition to demonstrating this effect in individual subjects. First, there was a significant overestimation towards the less rewarding option only for two out of four shapes (S3 and S4; two-sided sign test *P*=1 × 10^−6^ for S3 and *P*=6 × 10^−10^ for S4*, N*=37; Figs 3f and 4a). Note that the logLR associated with individual shapes indicates that when *P(B)*=0.75 (respectively, *P(B)*=0.25), S1 and S2 were presented more often when the blue (respectively, red) target was assigned with reward, whereas S3 and S4 were presented more often when the red (respectively, blue) target was assigned with reward (see Methods section). The overestimation of both S3 and S4 towards the less rewarding option was robust (present in 32 out of 37 subjects) and large (*m*=0.23±0.17), and was similar in strength for these shapes (two-sided rank-sum test *P*=0.7, *N*=37; Fig. 5a). Second, most subjects (25 out of 37) overestimated the predictive power of S3 to the extent that they assumed this shape was predictive of the less rewarding option (that is, larger than 0.5 for *P(B)*=0.25 and smaller than 0.5 for *P(B)*=0.75; one-sided sign test *P*=0.04, *N*=37; Figs 3f and 5a), while it was actually predictive of the more rewarding option. All these biases occurred while individuals were aware of the base rate (prior) as revealed by their answers to a short survey on completion of the task (Supplementary Fig. 1c).

Finally, the precise nature of our experimental design allows us to quantify the stochasticity in choice behaviour and estimation, measured by the *σ* values extracted from the PF of the choice session or the ePFs of the estimation session (Equation 3 in Methods section). Overall, the distribution of *σ* values was similar between the two groups of subjects who performed the experiment with different priors (two-sided rank-sum test *P*=0.4, 0.9, 0.2, and 0.6 for choice, one-shape, two-shape and four-shape estimates, respectively, *N*=37). However, we found a gradual increase in *σ* from one-shape to two-shape estimates (*σ*_1-shape_=0.49±0.28, *σ*_2-shape_=0.79±0.35) for 32 out of 37 subjects (two-sided rank-sum test *P*=3 × 10^−4^, *N*=37; Fig. 4d), and from two-shape to four-shape estimates (*σ*_4-shape_=1.22±0.50) for 33 out of 37 subjects (two-sided rank-sum test *P*=6 × 10^−5^, *N*=37; Fig. 4e). Interestingly, the amounts of stochasticity in choice behaviour (*σ*_choice_=1.11±0.57) and in four-shape estimates were statistically indistinguishable across all subjects (two-sided rank-sum test *P*=0.1, *N*=37; Fig. 4f). Therefore, the stochasticity in estimation increased (or equivalently the sensitivity to evidence, 1/*σ*, decreased) as the number of shapes used for estimation increased.

### Observed cognitive biases are not errors

All of the described systematic contradictory biases resulted from the unequal probability of reward for the two choice alternatives. In the case of equal prior (*P(B)*=0.5), we did not observe any systematic biases in choice behaviour or in the estimation (see the Supplementary Note 1 and Supplementary Figs 2 and 3a–c). However, similar to the experimental condition with unequal prior, we observed an increase in *σ* as the number of shapes used for estimation increased (Supplementary Fig. 3d–f).

The opposite patterns of bias for one-shape estimates and for choice behaviour, as well as the dependence of bias on the number of shapes used for estimation, are not errors and do not correspond to inconsistency between what subjects report and how they make decisions. To illustrate these points more clearly, we compared the SWOE for individual shapes with one-shape estimates given by each subject and found a similar pattern between the two (compare Fig. 3c,f, and see Supplementary Fig. 4c), indicating that subjects used these shapes to make decisions consistent with their estimations. That is, the same information was used for both choice and estimation. Another evidence for this comes from two sets of observations. First, the bias during the choice session was correlated with the bias for one-shape (Pearson's correlation, *r*=0.34, *N*=*35*, *P*=0.04), two-shape (Pearson's correlation*, r*=0.39, *N*=*35*, *P*=0.02) and four-shape estimates (Pearson's correlation*, r*=0.43, *N*=35, *P*=0.01; Fig. 5b). Second, although there was no correlation between *σ* during choice and estimation (Pearson's correlation, *r*=−0.22, 0.11 and −0.10, *P*=0.2, 0.5 and 0.6, for one-shape, two-shape and four-shape estimates, respectively, *N*=35), *σ* for one-shape and two-shapes estimates and for one-shape and four-shape estimates were strongly correlated (Pearson's correlation, *r*=0.63, *N*=*35*, *P*=10^−4^ and *r*=0.51, *N*=35, *P*=0.002, respectively; Fig. 5c). Overall, these results demonstrate that the opposite patterns of the biases for one-shape estimates and choice are not due to subjects relying on different sources of information or errors, and instead, caused by how posteriors for combinations of shapes are computed based on the information from individual shapes.

Therefore, our experimental results reveal that probabilistic inference does not follow a normative approach where likelihoods and prior information are combined optimally (or combined at all) to make decisions and to give estimates (see the last Results section for more proof). Instead, we argue that what is learned about each cue also contains information about the prior as these two pieces of information are not and cannot be separated during learning.

### Replicating data with a new model of probabilistic inference

**Figure 6:**
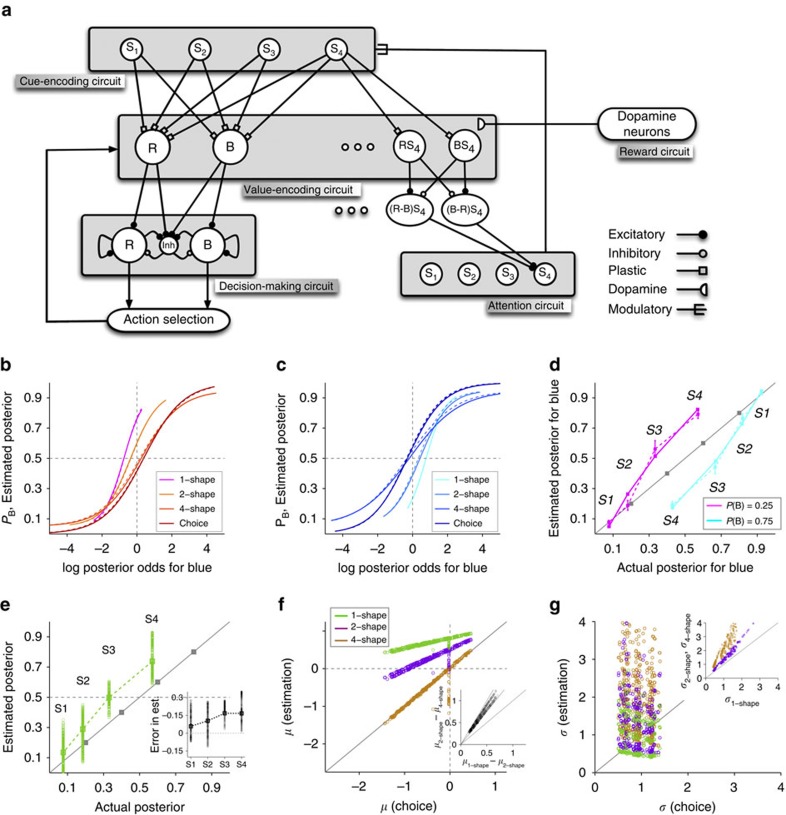
Schematic of the model and model's behaviour during the probabilistic inference task. (**a**) The schematic of the model and its five circuits (see Methods section). (**b**) Comparison of the ePFs and PF predicted by model (solid) and the fit of average experimental data (dashed) across subjects for whom the red target was more rewarding (the same curves as in Fig. 3a,d). (**c**) Comparison of the ePFs and PF predicted by the model (solid) and the fit of average experimental data (dashed) across subjects for whom the blue target was more rewarding (the same curves as in Fig. 3b,e). (**d**) Comparison of the estimated posteriors as a function of the actual posteriors for the model (solid) and for the experimental data (dashed). (**e**) The model's one-shape estimates towards the less rewarding option as a function of the actual posteriors, over a wide range of model's parameters (six parameters). The inset shows the difference between the estimated and actual posteriors, showing a bias towards the less rewarding option. (**f**) Model's estimation biases (relative to the more rewarding option) as a function of the bias in choice (relative to the more rewarding option) for the same set of parameters used in **e**. The inset shows the difference in the bias in two-shape and four-shape estimates as a function of the difference in the bias in one-shape and two-shape estimates. (**g**) The relationship between the model's stochasticity during estimation and choice. Conventions are the same as in Fig. 5.

We hypothesized that the cognitive biases observed during probabilistic inference are strongly influenced by interactions between reward-dependent learning, decision-making and attentional processes. To link these interactions to observed cognitive biases, we extended our previous model of probabilistic inference^14^. The previous model suggests that probabilistic inference can be performed by plastic synapses that learn cue–outcome associations via a stochastic, Hebbian plasticity rule modulated by the presence or the absence of reward. Specifically, on a given trial only synapses from active cue-encoding neurons onto active value-encoding neurons are potentiated if the choice on that trial was rewarded, or depressed if the choice was not rewarded (Supplementary Fig. 5). To extend this model, we incorporated three new components into the model (Fig. 6a; Methods section): (1) inclusion of a concave f-I response function for value-encoding neurons; (2) modulation of decision-making and learning processes by attentional selection; and (3) modulation of the learning rates by reward expectation.

The model simulated subjects' behaviour during both choice and estimation sessions and exhibited patterns of biases similar to those shown by our subjects, as demonstrated by the model's behaviour using one set of parameters replicating the average behaviour (Fig. 6b–d), and the model's behaviour using a wide range of parameters capturing inter-subject variability (Fig. 6e–g). More specifically, the model's choice behaviour was biased toward the more rewarding option, whereas one-shape estimates were biased towards the less rewarding option (Fig. 6b–f). Similar to experimental observations, the overestimation of the predictive power of individual shapes towards the less rewarding option was prominent for two of the four shapes, S3 and S4 (Fig. 6d). Moreover, with certain parameters the model overestimated the predictive power of S3 to the extent that it attributed this shape to be predictive of the less rewarding option, while it was actually predictive of the more rewarding option (Fig. 6e).

Similar to the experimental data, the model also showed a progression of estimation biases, starting from a bias towards the less rewarding option for one-shape estimates to a bias towards the more rewarding option for four-shape estimates (Fig. 6b,c,f). Finally, the sensitivity to evidence decreased as the number of shapes used for estimation increased (Fig. 6b,c,g). Interestingly, the above simulations using a wide range of parameters also exhibited correlations between the biases during estimations and choice (Fig. 6f) and between the sensitivity to evidence during estimations (Fig. 6g), similar to our experimental data (Fig. 5b,c). The qualitative similarity between our modelling and experimental results revealed the robustness of the model in capturing our novel experimental results. Overall, the model replicated all aspects of the experimental data and captured inter-subject variability using a wide range of parameters.

### Neural mechanisms underlying cognitive biases

**Figure 7:**
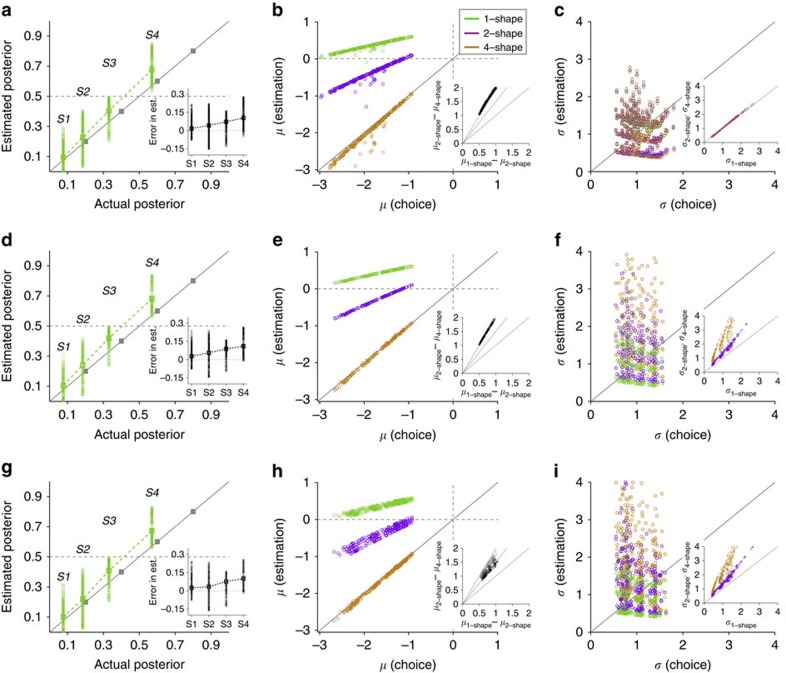
The behaviour of models lacking certain neural mechanisms. (**a**–**c**) Behaviour of the previous model with no additional components over a wide range of parameters (four parameters). (**d**–**f**) Behaviour of the previous model with a concave f-I response function for value-encoding neurons, over a wide range of parameters (four parameters). (**g**–**i**) Behaviour of the previous model with a concave f-I response function for value-encoding neurons and with modulation of learning and decision making by attentional selection, over a wide range of parameters (five parameters). Conventions are the same as in Figs 5 and 6e-g.

To demonstrate why all the three aforementioned components are necessary to capture the experimental data (that is, to avoid over fitting) and to gain further insights into the model's behaviour, we show next the results of simulating the experiment using our previous model^14^ (Supplementary Fig. 5) and models with successive addition of the three components. We found that the previous model learned evidence associated with each shape and showed a bias towards the more rewarding option (negative *μ*_choice_ values in Fig. 7b). Similar to the experimental data, however, the model's estimates were biased towards the less rewarding option when one shape was presented (Fig. 7a,b). This observation does not mean that information stored in each set of synapses is not biased towards the more rewarding option (which it is since most estimates are smaller than the evidence provided by the corresponding shape; Fig. 7a). Instead, it indicates that what is learned about individual shapes is not biased towards the more rewarding option as strongly as it should be optimally. This happens in the model since during the choice session, when the subject learns about shapes, four shapes together determines choice on each trial and, therefore, the bias in each set of synapses approximately constitutes one fourth of the overall bias required by the prior probability.

Moreover, as the number of shapes increased, the model's ePF shifted towards the more rewarding option (Fig. 7b). The shift happens because each set of synapses contains a piece of prior and, therefore, when more shapes are presented, the estimates are more biased towards the more rewarding option (that is, the choice alternative with higher prior). This also predicts that the shift between two-shape and four-shape estimates would be exactly twice the shift between one-shape and two-shape estimates (Fig. 7b, inset). These results were obtained for a wide range of the model's parameters (see Methods section for details).

Despite the previous model's success in performing the task, the model's behaviour did not match experimental data in three specific ways. First, the model predicted the sensitivity to evidence to be independent of the number of shapes used for estimation (Fig. 7c). Second, the model exhibited a shift in estimation bias that depended on the number of shapes used for estimation, but the shift between two-shape and four-shape estimates was twice the shift between one-shape and two-shape estimates (Fig. 7b). In contrast, this ratio was variable and about 1.5 on average in our experiment. Third, the model correctly estimated that shape S3 is predictive of the more rewarding option (Fig. 7a). In contrast, most subjects (25 out of 37) overestimated the predictive power of this shape to the extent that they attributed it to be predictive of the less rewarding option (Fig. 5a).

To show ‘pedagogically' how the aforementioned additional components can eliminate the above discrepancies and enable the model to replicate the experimental data, we successively incorporated the following components into the model. First, after considering a concave f-I response function for value-encoding neurons, we found that the sensitivity to evidence decreased as the number of shapes increased (Fig. 7d–f). A concave f-I curve (that is, a function with a monotonically decreasing derivative) has smaller slopes (gains) for higher values of input, which occurs when more shapes are presented. Second, introducing the possibility for learned evidence to control attentional deployment—ignoring the less predictive shapes (S2 and S3) when four shapes are presented (see Methods section)—enabled the model to reduce the difference in bias between two-shape and four-shape estimates (Fig. 7h, inset). As mentioned earlier, the size of the estimation bias is directly related to the number of shapes presented. Therefore, the possibility of ignoring less-informative shapes reduces the bias for four-shape estimates. During the choice session, such attentional modulation slightly reduced the bias towards the less rewarding option and increased the stochasticity in choice.

Third, by incorporating the modulation of DA-dependent plasticity by reward expectation for the chosen option (see Methods section), the model exhibited a larger overestimation for S3 such that it estimated this shape to be predictive of the less rewarding option (Fig. 6e). This increased overestimation happened because the potentiation rate for synapses from cue-encoding neurons selective for S3 onto value-encoding neurons selective for the less rewarding option was increased due to less-than-chance expectation of reward on this option (Equation 8). In contrast, the potentiation rate for synapses onto value-encoding neurons selective for the more rewarding option was reduced due to greater-than-chance expectation of reward on this option. This adjustment in the potentiation rate resulted in stronger synapses onto neurons encoding the value of the less rewarding option, leading to a greater overestimation of S3 (and of S4 for the same reason) towards that option (compare Figs 6e and 7g).

Overall, these results show that the inclusion of all aforementioned components is crucial for capturing nuances of our experimental observations, and shed light on the contribution of interactions between cognitive processes to probabilistic inference and related contradictory biases. First, choice is biased towards the more rewarding option since selection of that option is more frequently accompanied by reward; therefore, cue–outcomes associations stored at plastic synapses are biased towards that option. That is, what is learned about each shape contains a piece of prior information (compare estimated posteriors with evidence shown by grey squares in Fig. 6d,e). However, because choice is determined by the neural response invoked by presentation of four shapes, this bias is only a fraction (approximately ¼) of what prior information dictates. Therefore, information learned about individual shapes (posterior) is biased towards the less rewarding option. That is, similar to our experimental observations (Fig. 5a,b), what was learned about each shape was influenced by the prior probability, but not as strongly as prescribed by Bayes theorem. Second, as the number of shapes increases, multiple pieces of prior information bias the response of value-encoding neurons towards the more rewarding option, reducing the amount of bias for two-shape and four-shape estimates such that the latter bias becomes similar to the bias in choice (Fig. 6f).

Third, a concave f-I response function for value-encoding neurons causes those neurons to have smaller gains as their inputs increase; therefore, the estimation is less sensitive to the evidence. Fourth, modulation of decision making and learning by attentional selection reduces the impact of the prior probability encoded in each set of plastic synapses and the resulting shift in estimation. Finally, modulation of DA-dependent plasticity by reward expectation increases the bias towards the less rewarding option; on trials when this option is selected and rewarded, the potentiation rate is larger, increasing the bias of cue–outcome associations towards that option. Even though contradictory biases can occur due to decision-making and learning mechanisms alone, the interactions between decision and reward processes strongly affect these biases (compare Figs 7a–c and 6e–g).

### Comparisons with alternative models

Our model suggests that the computation of posteriors does not involve combining the prior probability and likelihoods. Instead, the computation is performed directly by learning cue–outcome associations via DA-dependent synaptic plasticity. These synaptically stored associations can approximate posteriors because learning is affected by the overall probability of reward on the two options (that is, prior). In essence, what is learned about a given shape is inherently a mixture of the prior probability and the likelihood provided by that shape, and this information is combined using the sum of the output currents through corresponding synapses when more than one shape is presented^14^. The observed overestimation of individual shapes and the dependence of estimation bias on the number of shapes provide strong evidence for our proposal. Nevertheless, to provide an additional proof for the basic assumptions of our model, we fit the behavioural data using three parameter-free models: the normative, heuristic and reduced circuit model (see Methods section). This parameter-free approach involved predicting two-shape and four-shape estimates based on one-shape estimates using different underlying assumptions. We used this approach for two reasons: (1) fitting results could be sensitive to the number of parameters used in different models; and (2) it is unclear how a normative model learns the task and, therefore, what the evidence provided by individual cues is in this model.

Briefly, the heuristic model assumes that subjects perform the probabilistic inference task by assigning a probability of predicting reward on red (or blue) for each shape, and using the average of the assigned probabilities for the presented shapes to make a decision or give an estimate. The normative model assumes that subjects separately learn the likelihood (equivalently, logLR) associated with individual shapes, as well as the prior probability for reward on red and blue. To make decisions or to give estimates, subjects optimally combine the prior probability and likelihoods from presented shapes using Bayes theorem. Finally, the reduced circuit model assumes that what is learned about each shape is an estimation of posteriors (that is, contains a piece of prior as in the neural circuit model), which is combined (in log space) to make decisions or provide estimates when more than one shape is presented.

**Figure 8:**
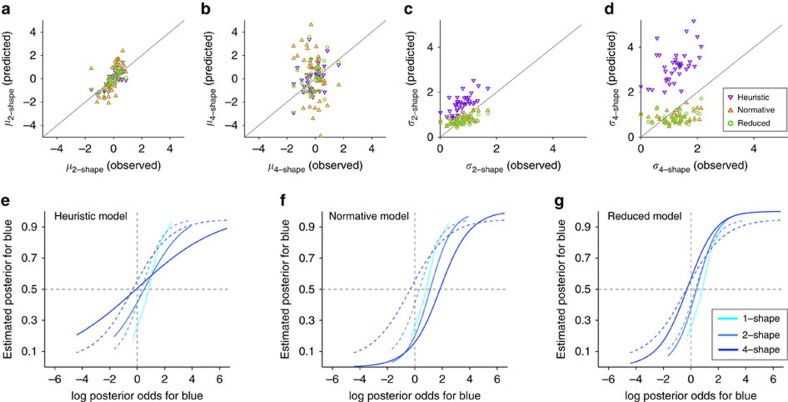
Alternative models fail to capture the experimental data. (**a**,**b**) The predicted bias in two-shape and four-shape estimates by two alternative models (heuristic and normative) and the reduced circuit model. Each point shows the prediction for one subject by a given model. (**c**,**d**) The predicted stochasticity in two-shape and four-shape estimates by the three alternative models. (**e**–**g**) Comparison of the ePFs predicted by the heuristic, normative, and reduced circuit models (solid) and the fit of average experimental data (dashed) across subjects for whom the blue target was more rewarding. The heuristic and normative models fail to predict either the bias or stochasticity, whereas the reduced circuit model provides a reasonable fit for both. Note that the averages for one-shape estimates are identical to those of experimental values because the same values were used for fitting.

Overall, the reduced circuit model provided the best fit (mean squared error (MSE)=16.6, 15.3 and 8.6 for the normative, heuristic and reduced circuit model, respectively). Figure 8 shows the results of predicted bias and stochasticity for two-shape and four-shape estimates for individual subjects (top panels), as well as the average predicted ePF for all subjects with *P(B)*=0.75 (bottom panels), using the three models. As expected, the heuristic model exhibited a strong decrease in the sensitivity to evidence (equivalently, increase in the stochasticity corresponding to larger *σ* values or shallower ePFs) as the number of shapes used for estimation increased (also see Supplementary Fig. 6).

The normative model predicted biases that changed with the number of shapes; however, the direction of these changes was the opposite of experimental observations. More specifically, two-shape and four-shape estimates were biased towards the less rewarding option, similar to one-shape estimates. This happens because in the normative model, the prior probability is similarly combined with evidence independent of the number of shapes used for estimation. Importantly, the discrepancy between this model's predicted biases and subjects' biases increased with the number of shapes (Fig. 8). In contrast, the reduced circuit model captured the experimental data and predicted a shift in estimation bias towards the more rewarding option as the number of shapes increased. Note that the observed discrepancy between the reduced circuit model and experimental observations can be attributed to lack of inclusion of a concave f-I response function, as well as lack of modulation of decision and learning processes by attention in this model (both of which are present in the neural circuit model). Nevertheless, the similarity between predicted and observed biases provides support for the main assumptions of our model regarding what is learned about each cue. That is, what is learned about each cue is a mixture of the likelihood and prior probability. As a result, when this information is combined to make decisions or provide estimates when more than one cue is presented, the inference exhibits biases that depend on the number of shapes.

## Discussion

Cognitive biases are often attributed to heuristics or limitations in cognitive processes^15^. Instead, we propose that these biases could depend on interactions between multiple cognitive processes and therefore, provide a window into better understanding cognition. To reveal neural mechanisms underlying cognitive biases during probabilistic inference, we measured behaviour during a modified version of the so-called weather prediction task and simulated this behaviour using a biophysically plausible computational model. Specifically, we investigated similarities and differences between what is learned implicitly (reflected in choice) and what is reported explicitly during inference within individual subjects. Our experimental results showed that when alternative options were not equally rewarding, subjects' choice behaviour was biased towards the more rewarding option, whereas their estimates for individual shapes that more often preceded reward on the less rewarding option (that is, rare outcome) were biased towards that option (base-rate neglect). Moreover, biases and accuracy of estimation systematically depended on the number of shapes used to provide those estimates, contradicting any normative accounts of probabilistic reasoning.

The base-rate effect has been shown before^1^,^16^,^20^, but our experiment is the first to demonstrate this effect within individual subjects, even though the same individuals learned the base rate of reward for each option (that is, prior) and biased their choice behaviour towards the more rewarding option. Although connectionist models have previously accounted for the overall pattern of base-rate neglect, their proposed mechanisms require access to all connection weights in the network^1^,^20^,^21^. Interestingly, in a set of experiments designed to understand base-rate effects, Estes *et al*.^20^ concluded that none of the considered models could explain how subjects' awareness of base rate failed to affect their performance during test trials. In contrast, our biophysically inspired model accounts for the exact pattern of base-rate effect based on a simultaneous learning of all cue–outcome associations via DA-dependent plasticity modulated by reward expectation, and modulation of decision and learning processes by attention.

The fundamental outcome of the synaptic plasticity rule of our model is that evidence learned about individual cues is contaminated by the prior probability. This predicts that biases in estimated posteriors systematically depend on the number of cues used for estimation (which was observed in our experiment), contradicting any notion of a normative calculation of posteriors. Note that we did not add a separate set of synapses to our model to learn the prior probability because such a component would equally bias choice and estimation towards the more rewarding option. Overall, our results do not suggest that the brain fails to track the prior probability and instead, indicate that the computation of posteriors does not involve combining prior and likelihoods.

Furthermore, the model assumes that information stored in plastic synapses can be accessed to provide explicit estimations about the predictive power of individual cues and/or their combination. This assumption is supported by the strong correlation between the SWOEs extracted from choice behaviour and one-shape, two-shape and four-shape estimates (Supplementary Fig. 4), as well as the similar patterns of biases in the SWOEs and one-shape estimates (Fig. 3c,f). These findings indicate that to give estimates, subjects accessed the same information that they learned implicitly to make decisions. However, because both decision making and estimation are determined by the output of value-encoding neurons, the f-I response function of these neurons influences these processes as supported by an increase in the stochasticity as the number of shapes used for estimation increases. In addition, we tested the possibility of using a simple averaging strategy for performing the task and found that such a strategy is incompatible with our experimental data. Therefore, complementing our previous proposal for synaptic substrates of probabilistic inference^14^, the current study identifies additional neural mechanisms.

Previous works using the weather prediction task have revealed important aspects of human learning and memory^2^,^3^,^4^,^5^,^6^,^7^,^8^,^10^,^11^,^12^,^13^, namely, the existence of two memory/learning systems that contribute to probabilistic inference at different time points during learning. Our modelling results shed light on the differential role of brain areas previously implicated in these two systems^6^,^13^. Specifically, our results highlight the importance of neurons with mixed selectivity^22^ for an action and a shape, since such neurons (for example, in medial temporal lobe) could quickly encode reward value and guide attentional selection to allow decision making based on a single cue early in the task^6^,^8^,^11^. Moreover, our results suggest that slower learning in value-encoding neurons selective for action alone are necessary for proper learning, which explains why areas containing such neurons (for example, basal ganglia) become more active later in the task^6^. Considering the crucial role of DA in synaptic plasticity^23^ underlying any type of value encoding^24^, the model also accounts for Parkinson's patients' slowness both early in learning and later in adopting optimal strategy^4^, due to their impaired DA system^25^. Interestingly, a recent model of category learning was able to explain the pattern of learning in Parkinson's patients based on interactions between prefrontal and striatal areas mediating different types of learning^26^.

Even though the contribution of attention to cognitive biases has not been extensively explored^26^, most models of multi-attribute, multi-alternative choice assume a role for attention to explain various aspects of human choice behaviour, such as context-dependent effects. For example, the elimination by aspects model^27^, the decision field theory^28^,^29^, and the leaky competing accumulator model^30^ all assume that attentional selection determines which of many attributes are attended to, consequently affecting decision making processes at a given point in time. More recently, this idea has been extended to choice related to food items, assuming that fixation guides the comparison process^31^. However, these models do not consider the effect of reward feedback and focus only on how decision making is affected by attentional selection in the absence of learning.

We showed that modulation of DA-dependent synaptic plasticity by reward expectation is crucial for capturing the patterns of overestimation and base-rate neglect. Although this assumption resembles the reward prediction error (RPE), it only applies to the DA signal when the reward is present (the DA signal in the absence of reward is always set to zero); that is, when outcomes are better than expected. This assumption is not only supported experimentally^32^, but also proves more plausible than the mediation of negative RPE by below-baseline activity of DA neurons in the absence of reward. In contrast, modulation of the DA signal by reward expectation for rewarded trials resembles the unsigned RPE^33^,^34^, which has been linked to attentional learning models^35^.

The biophysically inspired nature of our model allows us to make further behavioural and neural predictions that can be tested in future experiments. First, the model suggests that the prior probability cannot be separated from evidence when both have to be learned through reward feedback, and, therefore, neurons or areas representing the reward value of individual shapes could show a modulation by the prior probability as well. This modulation can be measured and tested by varying the prior without changing the evidence associated with individual shapes. Second, sensory neurons representing shapes could show attentional modulation, while the evidence for shapes is being learned. Importantly, the degree of such modulation is correlated with the amount of shift in the bias between two-shape and four-shape estimates. Third, the model predicts that overestimation of individual shapes towards the less rewarding option should correlate with the influence of reward expectation on the learning rates, which can be measured using reward expectation as a regressor for neural response. In addition, one could use data from human subjects and look for within-subject correlation between measures of dopaminergic activity (for example, reward reactivity, gene expression) and the amount of overestimation for individual shapes. Finally, the decision-making circuit relies on the difference between the inputs to its selective populations (differential input) to determine a choice, and the reaction times are inversely proportional to this differential input. This means that during the choice session, decisions are faster for trials on which the evidence provided by a combination of shapes is farther from zero. Moreover, the model assumes that estimation relies on the same sets of synapses used for making decisions. Therefore, one prediction would be that reaction times for one-shape estimates for S3 and S4 is inversely correlated with the deviation of the estimation from 0.5, which could be tested, considering the large variability in subjects' estimations.

Altogether, our results suggest that probabilistic inference is performed by directly estimating posteriors and not by combining likelihoods and prior, resulting in deviations from the normative accounts of inference (that is, cognitive biases). Moreover, these cognitive biases are influenced by interactions between reward-dependent learning, decision-making and attentional processes that dynamically determine probabilistic inference.

## Methods

### Subjects

A total of 60 (34 female) Dartmouth college undergraduate students participated in this experiment. Eleven subjects were excluded from data presented here for the following reasons: five subjects for not learning the task properly, defined as having the stochasticity during choice or estimation session three s.d. larger than the mean of all subjects; six subjects for giving three (out of four) or more inconsistent estimates for blue and red targets during one-shape estimation (two of whom could also be excluded based on the first criterion). The consistency criterion during one-shape estimation was used as a test for trusting all estimates, since for two-shape and four-shape estimates either the blue or red target was probed for each combination of shapes. The results from 37 (21 female) subjects who performed the main experiment are reported here, 19 of whom performed the experiment with the prior probability of the blue target being rewarded, *P(B)*, equal to 0.75. The remaining 18 subjects performed the experiment with *P(B)*=0.25. For the control experiment, we recruited 12 subjects (6 female) and ran the experiment with equal probabilities of reward on the red and blue targets (equal prior, *P(B)*=0.5). All subjects were naive to the experiment and the goal of our study.

### Experimental procedure

After receiving and signing the consent form, subjects received instructions and then played a few mock trials to learn about the two sessions of the experiment (choice and estimation), as well as different types of trials, timing of trials and how to submit their responses using a keyboard. This was followed by a training phase consisting of 120 choice trials in which subjects learned about the predictive power of 4 randomly assigned shapes (from a set of 10, Fig. 1) through reward feedback (with *P(B)*=0.5), followed by 18 estimation trials. During the choice session of the training phase, various combinations of two shapes (chosen randomly from a set of four shapes with replacement) were presented together on each trial. During the estimation session of the training phase, subjects provided their estimates about the predictive power of individual shapes and two-shape combinations of shapes.

The training phase was followed by the main experiment during which subjects performed a similar task using four new shapes. In the main experiment, however, various combinations of four shapes were presented on each trial during the choice session. During the estimation session, subjects provided their estimates about the predictive power of individual shapes, two-shape and four-shape combinations of shapes. The main experiment consisted of 212 choice trials (divided into 4 equal blocks of 53 trials) followed by 53 estimation trials.

The subjects were informed that their payment in the experiment depended on the number of reward bars they collected, as well as how closely their estimates resembled the actual values. Moreover, the subjects were told that presented shapes on each trial predicted reward on the red or blue targets, and the rule used for assigning reward did not depend on the location of the shapes. Subjects were randomly assigned to experimental conditions with an unequal prior probability (*P(B)*=0.25 or *P(B)*=0.75).

Overall, we used 10 unique, geometric shapes as visual cues in our experiment (Fig. 1a). For each subject, 8 (4 for training and 4 for the main experiment) out of 10 shapes were randomly selected and assigned with one of the four log likelihood ratios (logLR) towards the blue target:


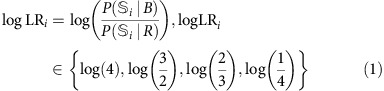


where 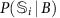 represents the likelihood that shape *i* was presented, given the blue target was assigned with reward. In our analysis, we refer to shapes assigned with above logLR values in descending order, S1 to S4 for *P(B)*=0.5 and *P(B)*=0.75, and S4 to S1 for *P(B)*=0.25. To determine which shapes would be presented on each trial of the experiment, we first randomly assigned reward to one of the two options according to the prior probability, *P(B)* (equal to 0.25 or 0.75 for subjects in the main experiment, and 0.5 for training and for the control subjects). Subsequently, depending on the assigned reward on a given trial, the computer program used the logLRs to determine which of four shapes should be present. This procedure was repeated two (for training) or four times (for the main experiment) to assign all shapes on each trial. Note that in the case of unequal prior, four shapes were not presented equally often.

During each trial of the choice session, subjects were presented with two (during the training phase) or four shapes (during the main experiment) and chose between the two colour targets by pressing the letters ‘F' or ‘J' to select the left or right target, respectively. A circle appeared around the chosen target, followed by the presentation of reward feedback (correct/incorrect) and an update of the reward bar that showed recent accumulation of reward points (Fig. 1b). When the reward bar reached a certain length, its colour changed to gold and a message, ‘You earn 25 cents' was displayed above the bar, followed by resetting the length of reward bar to zero.

During each trial of the estimation session, subjects estimated the probability that the red or blue target would be rewarded given a shape or a combination of shapes (Fig. 1c). This session started with 8 trials of one-shape estimates (4 for blue and 4 for red) followed by 10 unique two-shape estimates and 35 unique four-shape estimates. The two-shape and four-shape estimates were obtained for either the red or blue target to reduce the overall length of the experiment. In contrast, one-shape estimates were obtained twice for each shape (once for red and once for blue) and a discrepancy between the two numbers was used to detect subjects who either did not understand the estimation procedure or did not pay attention to the colour of the target.

After completion of the experiment, the subjects were asked to write down the overall probability that the blue target was rewarded during the training as well as during the main experiment. All experimental procedures were approved by the Committee for the Protection of Human Participants of Dartmouth College, and a written consent was obtained from each subject before participating in the experiment.

### Data analysis

To quantify each subject's behaviour during the choice session, we computed the PF by calculating the probability that the blue target was selected for a given combination of shapes as a function of the log posterior odds for that combination. Using Bayes theorem, the log posterior odds for reward being on blue versus red is equal to


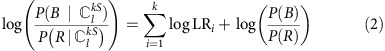


where 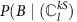 (respectively, 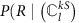) is the posterior probability that the blue (respectively, red) target was assigned with reward given a combination of shapes, 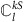, consisting of *k* shapes, was presented on a given trial. We fit this PF with a sigmoid function to extract *μ* (the log posterior odds at which two choice alternatives are selected equally or the indifference point) that measures the subject's bias, as well as *σ* that measures stochasticity in the subject's choice behaviour (inversely proportional to the sensitivity to evidence):


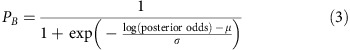


where *P*_*B*_ is the probability of choosing the blue target. Note that based on Equation 2, the optimal behaviour is obtained with *μ*=0 and very small values of *σ*. To quantify behaviour in the estimation session, we computed the ePF that show the relationship between estimations provided by the subject and the actual log posterior odds for each shape or combination of shapes. A fitting procedure similar to one used for the PF was applied for estimating *μ* and *σ* for the ePF.

Moreover, we used a logistic regression model to extract the influence of individual shapes on choice behaviour:


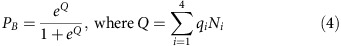


where *N*_*i*_ is the number of appearances of shape *i* in a given combination. We call the regression coefficients (*q*_*i*_'s) from this fit the SWOE for individual shapes. Note that we did not include a bias term here because there is a fixed number of shapes on each trial, and therefore, an additional bias term would cause degeneracy in fitting (that is, the sum of regression coefficient can absorb the bias term). Extracted SWOEs from subjects showed that subjects learned most of the information after around 60 trials (Supplementary Fig. 7). Therefore, we limited our data analysis for the choice session to the last three (out of four) 53-trial blocks.

### Description of the model

The model is an extended version of our previous biophysically based model of probabilistic decision making^14^. More specifically, we incorporated three new components into the previous model. First, because information stored in plastic synapses onto value-encoding neurons is accessible only via activating postsynaptic neurons, the response properties of these neurons could influence probabilistic inference. Therefore, instead of a linear f-I response function, we incorporated a more realistic concave function for value-encoding neurons. Second, we assumed that existing information about the predictive power of cues (stored at the synaptic level) affects attentional selection to determine which cues are registered on a given trial and used to make a decision. Later in the trial when reward feedback is received, only neurons selective to the registered/attended cues are active and therefore, only synapses from those neurons onto value-encoding neurons selective to the chosen target are updated. Finally, we assumed that DA-dependent plasticity is modulated by the expectation of reward on the chosen option on each trial. For simplicity and because here we were not concerned with neural activity in different parts of the brain, we used a mean-field reduction of the decision-making and attention circuits to simulate the behaviour in the probabilistic inference experiment (see below).

The model consists of five circuits (Fig. 6a): cue-encoding, value-encoding, decision-making, attention and reward. The cue-encoding circuit contains sensory neurons that are selective for individual visual cues (shapes), and can be located in the inferotemporal cortex, where shape-selective neurons have been found^36^,^37^. The cue-encoding neurons project to the value-encoding circuit (Fig. 6a). The value-encoding circuit contains two pools of neurons that represent the reward value of the two alternative responses (action values), and separate pools of neurons that encode the reward value of an alternative response associated with a given shape (selective to both action and shape). The former pools contribute to decision making, whereas the latter contribute to attentional selection (see below). These neurons acquire such representation through their afferent plastic synapses that undergo reward-dependent Hebbian modifications^14^,^38^,^39^,^40^ (see Learning rule). Moreover, we assumed that the output of value-encoding neurons is determined by the strength of plastic synapses as well as the f-I response function of these neurons. Neurons encoding action values can be found in the basal ganglia^41^,^42^,^43^ or frontal cortices such as the anterior cingulate^44^ or dorsolateral prefrontal cortex^45^,^46^. Value-encoding neurons that are selective to action and shape can be found in the medial temporal lobe or dorsal frontal cortex^6^,^47^.

The decision-making circuit receives inputs from neurons encoding action values (Fig. 6a). This circuit contains two competing neural pools that are selective for alternative responses (*B* and *R*, corresponding to blue and red targets, respectively), and an inhibitory pool of neurons. As we have shown before^14^,^38^,^39^, the choice on each trial is stochastic due to neural fluctuations but the probability of choice is a sigmoid function of the difference in inputs to the two selective pools. The responses of cue-encoding neurons are similar; therefore, unless neural activity is modulated by attention (see below), the only factor that differentiates the inputs to two selective pools of decision circuit is the strength of plastic synapses between cue-encoding neurons and value-encoding neurons. As a result, decision making is simulated by first calculating the choice probability using the synaptic strengths onto value-encoding neurons:


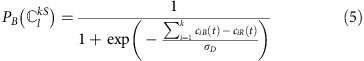


where (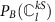) is the probability of selecting *B* given a combination of shapes 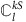 that is presented on trial *t*, *1*/*σ*_*D*_ quantifies the sensitivity of the decision network to the difference in its inputs, *c*_*iB*_(t) represents the average strength of synapses (that is, fraction of synapses in the strong state) between neurons encoding shape *i* to neurons encoding the value of the blue target, and the sum is over all shapes presented in that combination (the current evoked by a repeated shape is multiplied by the number of repetition). Importantly, the convergence from cue-encoding neurons enables value-encoding neurons to combine information from various cues presented on a given trial. Moreover, to give estimates, the overall output from the value-encoding neurons evoked by the presentation of a shape or combination of shapes is passed through an equation similar to Equation 5 but with *σ*_*E*_.

The attention circuit also receives inputs from value-encoding neurons that are selective to action and shape (Fig. 6a). We assumed that attentional deployment depends on how informative a shape is independent of which target the shape predicts. Therefore, the difference between the outputs of value-encoding neurons that are selective to action and shape (see (R-B)S4 and (B-R)S4 pools in Fig.6a) drives neurons in the attention circuit to generate a signal for attending/registering (respectively, ignoring) the informative (respectively, less informative) shapes using a competitive process. Only synapses from attended shapes are updated when reward feedback is received (see Learning rule). For simplicity, here we assumed that on a fraction of trials, only one of the two less-informative shapes is ignored throughout a trial (with the probability *p*_ign_), but this mechanism could be generalized to include the possibility of ignoring any shapes with a probability that depends on the predictive power of that shape. Finally, the reward circuit signals the presence or absence of reward at the end of each trial. We assumed that the absence of reward is signalled with no DA release (and therefore, cannot be modulated by reward expectation), whereas the presence of reward is signalled by DA release modulated by the expectation of reward on the chosen target (see Learning rule).

### Learning rule

The inputs to the decision circuit are determined by the activity of sensory neural pools encoding the presented shapes, and by the strength of plastic synapses from these populations onto value-encoding populations. Similar to our previous work^14^,^38^,^39^, we assumed that these plastic synapses are binary (that is, they only have two stable states). The average strength of these synapses can be defined as the fraction of synapses in the potentiated state, denoted by *c*_*iB*_ and *c*_*iR*_ (for synapses from cue-encoding neurons selective for shape *i* onto value-encoding neurons selective for the blue and red targets, respectively).

At the end of each trial, plastic synapses were modified according to a stochastic, reward-dependent, Hebbian learning rule. First, Hebbian plasticity required a high level of activity in both pre- and post-synaptic neurons so only synapses from active cue-encoding neurons onto the value-encoding neurons selective for the chosen target were modified. Neurons encoding the reward value of a given shape are assumed active only if that shape was registered/attended. We assumed value-encoding neurons selective to shape and action behave similarly. Second, depending on the outcome (reward or no reward) on a given trial, plastic synapses were either potentiated or depressed. However, the potentiation rate depends on the concentration of DA, which itself depends on reward expectation (see below). In the absence of reward, DA neurons fire at a very low rate (DA concentration is very low) and therefore, modulation of the depression rate by reward expectation is not meaningful. Third, these synaptic modifications occur stochastically.

Specifically, at the end of an unrewarded trial where the blue target was selected, synaptic strengths (that is, fraction of synapses in the strong state) for all registered/attended shapes onto value-encoding neurons selective for this target were updated as (assuming there is a large number of synapses to consider synaptic strengths as continuous variables)


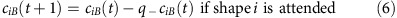


where *q*_*−*_ is the depression rate. On rewarded trials, synaptic strengths are updated as follows


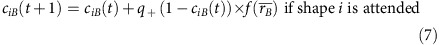


where *q*_*+*_ is the potentiation rate, and 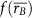 incorporates the influence of reward expectation for the selected target on DA release and on synaptic plasticity (see below). All other plastic synapses (synapses from neurons selective for unattended/ignored shapes, or synapses onto neurons selective for the unchosen target) remain the same. Here we have assumed that the learning rates (*q*_*p*_ and *q*_*d*_) do not depend on the number of repetitions of a presented shape, but qualitatively similar results were obtained if the learning rates monotonically increase with this number.

The effect of reward expectation on the potentiation rate (via changes in DA release) is computed as:


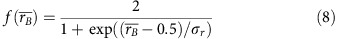


where 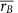 denotes the local average reward (computed by other sets of synapses described below) when the blue target was chosen, and *1/σ*_*r*_ determines the sensitivity of DA release to reward expectations. The local average of reward on the blue target (and similarly for the red target) is updated as:


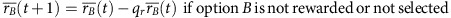



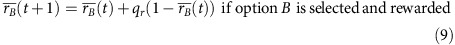


Because the chance level of reward for binary choices is 0.5, this value is used as a baseline in Equation 8, and *q*_*r*_ was set to 0.1 in all simulations. Synapses onto value-encoding neurons selective for both action and shape undergo a similar learning rule, but with larger learning rates. The faster learning allows the attention circuit to selectively process individual shapes early in the experiment.

### Models parameters

For simulations shown in Figures 6 and 7 we used a wide range of parameters to show the robustness of our results and capture inter-subject variability. For those simulations, the model's behaviour was measured over 100,000 simulated trials to accurately capture the average behaviour for a given set of parameters. For simulations of the original model (Fig. 7a–c), we sampled all combinations of parameters (total four parameters) from the following values with the constraint that *q*_*p*_<2.5*q*_*d*_:*q*_*p*_=[0.02, 0.04, 0.06, 0.08, 0.10]; *q*_*d*_=[0.02, 0.04, 0.06, 0.08, 0.10, 0.12]; *σ*_*D*_=[0.05, 0.10, 0.20, 0.30]; *σ*_*E*_=[0.10, 0.20, 0.30]. We limited the range to the above values to avoid extreme biases towards the more rewarding option during the choice session, which occurs when the learning rates are greater than 0.12, or when *q*_*p*_ and *q*_*d*_are comparable in magnitude. For simulations of the original model with the additional concave f-I response function for the value-encoding neurons (Fig. 7d–f), we used an f-I curve function that captures the average behavioural data and sampled all combinations of other parameters (total seven parameters) from the following values: *q*_*p*_=[0.02, 0.04, 0.06]; *q*_*d*_=[0.04, 0.06, 0.08, 0.10, 0.12]; *σ*_*D*_=[0.05, 0.10, 0.20, 0.30]; and *σ*_*E*_=[0.05, 0.10, 0.15]. For simulations of the model which has all components of the full model except the modulation of the potentiation rate by reward expectation (Fig. 7g–i), we sampled all combinations of parameters (total eight parameters) from the following values: *q*_*p*_=[0.02, 0.04, 0.06]; *q*_*d*_=[0.04, 0.06, 0.08, 0.10, 0.12]; *σ*_*D*_=[0.05, 0.10, 0.20, 0.30]; *σ*_*E*_=[0.05, 0.10, 0.15]; and *p*_ign_=[0.2, 0.4, 0.6, 0.8]. For simulations of the complete model, we fixed *q*_*r*_ at 0.1 and sampled all combinations of parameters (total ten parameters) from the following values: *q*_*p*_=[0.02, 0.04, 0.06]; *q*_*d*_=[0.04, 0.06, 0.08, 0.10, 0.12]; *σ*_*D*_=[0.05, 0.15, 0.25]; *σ*_*E*_=[0.05, 0.10, 0.15]; *p*_ign_=[0.3, 0.6]; and *σ*_*r*_=[0.1, 0.2, 0.3, 0.4, 0.5].

### Heuristic model

The heuristic model assumes that subjects perform probabilistic inference by assigning a probability of predicting reward on the blue (or red) target for each shape and updating these probabilities using reward feedback. Specifically, subjects use the average of the assigned probabilities for the presented shapes to estimate the predictive power of a combination of shapes. To make a choice, we assumed that the average of reward probabilities assigned to the presented shapes are mapped to a sigmoid function to allow a wide range of behaviour (from probability matching to optimal and so on)


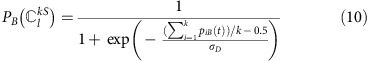


where *p*_*iB*_ is the probability of reward on blue if shape *i* is presented alone, and *σ*_*D*_ is a model parameter. The assigned probabilities are updated after receiving feedback on every trial using equations similar to Equations 6 and 7, where synaptic strengths (*c*_*iB*_) are replaced with reward probabilities (*p*_*iB*_). Similar to our neural circuit model, we assumed that the potentiation rate could be modulated by reward expectation based on Equation 8 to reduce a strong bias in choice towards the more rewarding option, which would otherwise occur. For simulations of the heuristic model (Supplementary Fig. 6), we fixed *q*_*r*_ at 0.1 and sampled all combinations of parameters (total six parameters) from the following values: *q*_*p*_=[0.02, 0.04, 0.06]; *q*_*d*_=[0.02, 0.04, 0.06, 0.08, 0.10, 0.12]; *σ*_*D*_=[0.025, 0.05, 0.10, 0.15]; *σ*_*r*_=[0.05, 0.10, 0.15].

### Parameter-free fitting

To test the basic assumptions of our model regarding what information is learned about individual shapes and how information from multiple shapes is combined to make decisions and give estimates, we fit the experimental data using three models: the heuristic, normative, and reduced circuit model. More specifically, we used one-shape estimates given by individual subjects to predict their two-shape and four-shape estimates. We used this fitting method because it is parameter-free and avoids any assumptions about learning in the normative model.

First, the heuristic model assumes that subjects perform probabilistic inference tasks by assigning a probability of predicting reward on the blue (or red) target for each shape, and using the average of the assigned probabilities for the presented shapes to give an estimate. To fit the data using the heuristic model, we predicted two-shape and four-shape estimates for each subject by simply averaging one-shape estimates for the presented shapes on a given trial (including repetition).

Second, the normative model assumes that subjects separately learn the likelihood (equivalently, logLR) associated with individual shapes, as well as the prior probability for reward on red and blue. Moreover, to make decisions or give estimates, subjects optimally combine the prior probability and likelihoods from presented shapes using Bayes theorem. Although it is unclear how such learning could be performed in the brain (or even by a Bayesian learner) and whether these computations can be done without any bias, we wanted to test predictions of this model regarding changes in estimation as the number of shapes used for estimation increased. To calculate predictions for the normative model, we first used the prior probability and one-shape estimates provided by a given subject to extract the logLR associated with each shape for that subject. Following this, two-shape and four-shape estimates were predicted by summating the log prior odds and logLRs of the presented shapes to obtain the log posterior odds, and then converting the resulting log posterior odds to posteriors.

Finally, we used the main assumption of our model to arrive at the reduced circuit model. That is, what is learned about each shape is a mixture of prior and evidence and this information is used to provide estimates about a combination of shapes. To compute predictions for the reduced circuit model, we first calculated the log posterior odds for individual shapes using one-shape estimates for a given subject. The two-shape and four-shape estimates were calculated by adding log posterior odds for shapes presented in a given combination, which were then converted to posteriors.

## Additional information

**How to cite this article:** Soltani, A. *et al*. Neural substrates of cognitive biases during probabilistic inference. *Nat. Commun.* 7:11393 doi: 10.1038/ncomms11393 (2016).

## Supplementary Material

Supplementary Information Supplementary Figures 1-7 and Supplementary Note 1
