# Supplementary material for: Neural substrates of cognitive biases during probabilistic inference
Source: Nat Commun. 2016 Apr 26;7:11393. doi: 10.1038/ncomms11393 (PMC4853436; doi:10.1038/ncomms11393)
Supplement: Supplementary Information — Supplementary Figures 1-7 and Supplementary Note 1 [file ncomms11393-s1.pdf]

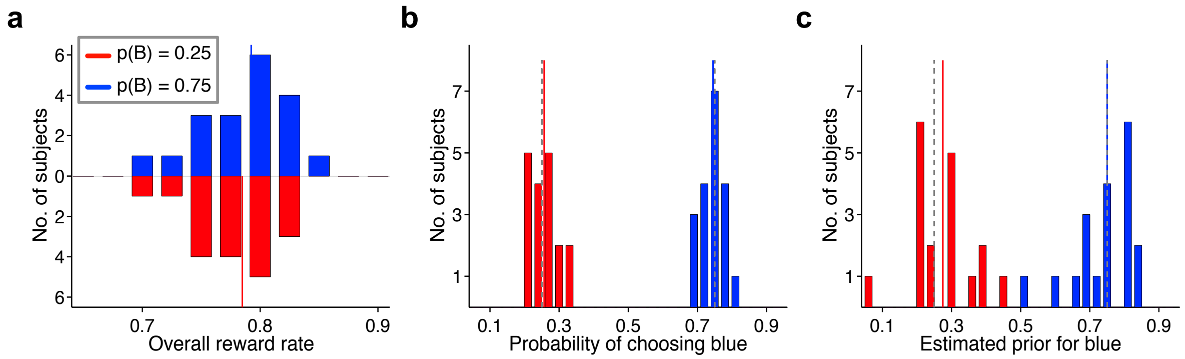

Supplementary Figure 1 | Overall performance and estimation of the prior probability by individual subjects. (**a**) Distributions of the overall reward rate for the two groups of subjects. The red and blue distributions correspond to the reward rate for subjects for whom the red or blue target was more rewarding, respectively. The color lines show the median for each set of subjects. (**b**) Distributions of the overall probability of selecting the blue target for the two groups of subjects. The gray dashed lines are centered at 0.2 and 0.8. Each group selected the more rewarding target close to the prior probability for that group. (**c**) Distributions of the estimated prior probability for the two groups of subjects. The median of each group falls close to the prior probability for that group. Conventions are the same as in **b**.

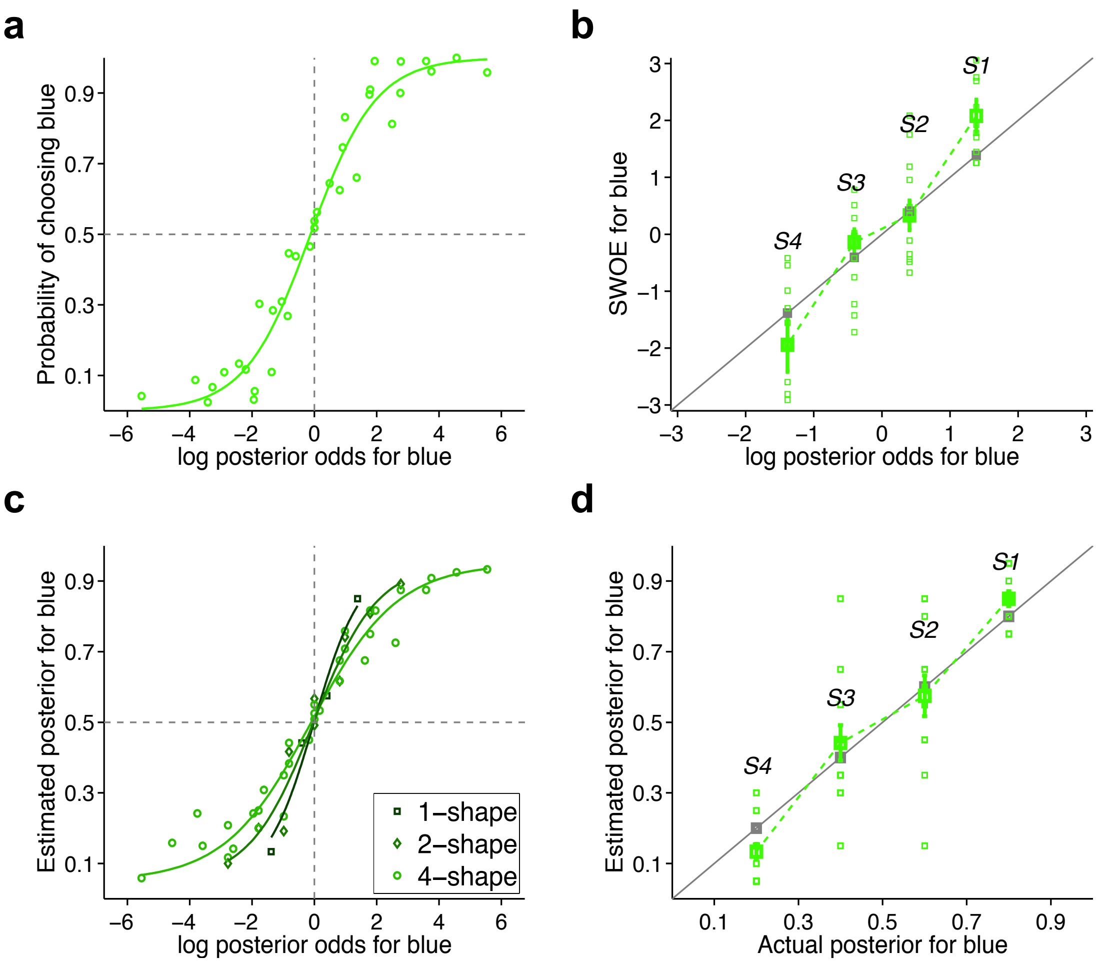

Supplementary Figure 2 | Average choice and estimation behavior across all subjects during the control experiment. (**a**) The average PF function over all subjects during the control condition. (**b**) The SWOEs extracted from the choice behavior of individual subjects are plotted against the actual log posterior odds for each shape (empty symbols). The filled symbols show the average SWOE for each shape across all subjects, and the error bars are the s.e.m. The dashed lines are only to guide the eyes, the gray solid line is the diagonal line, and gray squares show the logLR associated with each shape. (**c**) The average ePF over all subjects, for 1-shape, 2-shape, and 4-shape estimates. (**d**) Estimated posteriors for individual shapes as a function of the actual posteriors, provided by each subject. Gray squares show the evidence associated with each shape. Other conventions are the same as in **b**.

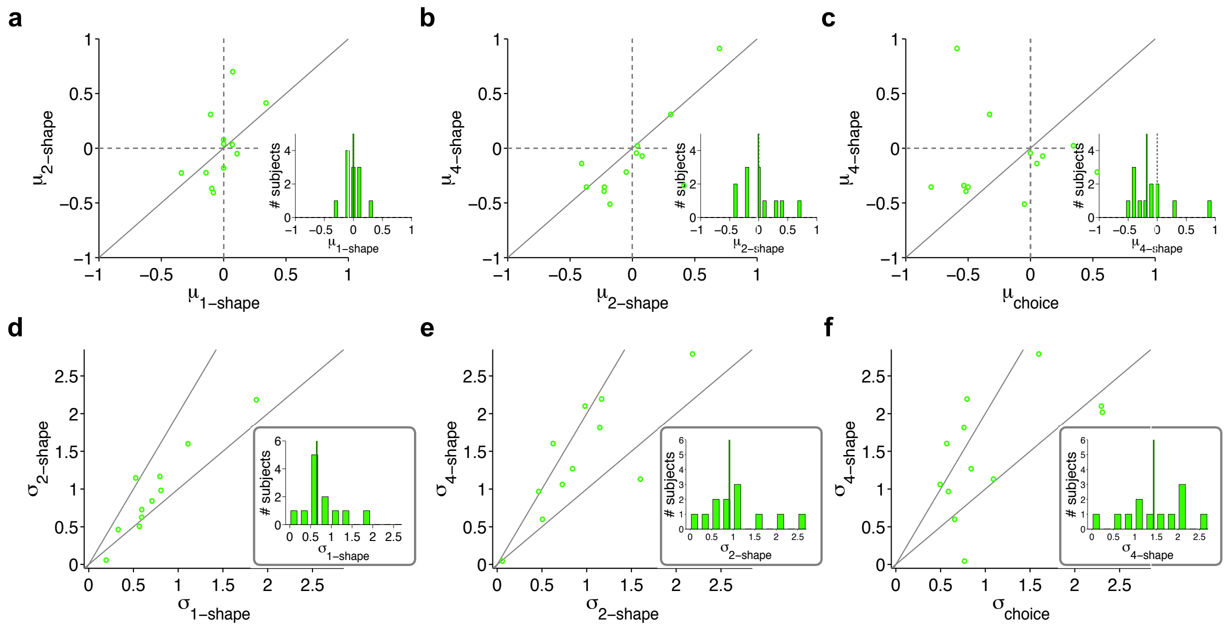

Supplementary Figure 3 | Within-subject changes in the bias and sensitivity to evidence as a function of the number of shapes during the control experiment. (**a**) Plotted are the biases for 2-shape estimates ($\mu_{2\text{-}shape}$) versus the biases for 1-shape estimates ($\mu_{1\text{-}shape}$) for individual subjects. The histogram plots the frequency of subjects with a given bias for 1-shape estimates, and the solid line is the median for each set of subjects. (**b**) The same as in **a**, but for bias for 4-shape estimates ($\mu_{4\text{-}shape}$) versus the bias for 2-shape estimates ($\mu_{2\text{-}shape}$). (**c**) The same as in **a**, but for the bias for 4-shape estimates ($\mu_{4\text{-}shape}$) versus the bias during the choice session (μ_{choice}) (**d-f**) Comparison of the stochasticity in choice and estimations, measured by σ from the ePF or PF for individual subjects. The lower and higher dashed lines show the lines with slope 1 and 2, respectively. The inset shows the histogram of σ values for 1-shape to 4-shape estimates, and the solid lines show the median for a given distribution.

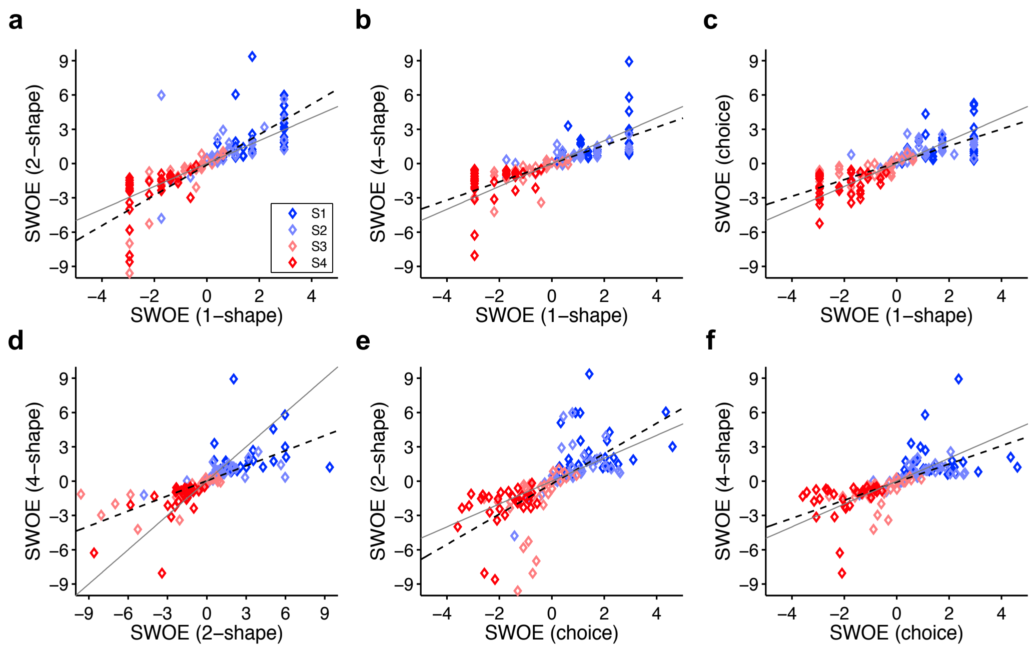

Supplementary Figure 4 | Correlation between the SWOE calculated from choice and from estimations provided by individual subjects. Each point shows the SWOE for a given shape for a given subject. The black dashed lines show the linear fit for each set of data, and the diagonal lines are shown in solid gray. Panels **a-b** show a strong correlation between the SWOE for 2-shape and 1-shape estimates (Pearson correlation, $r = 0.75$ and 0.88, $N = 37$, $P < 10^{-10}$, for $p(B) = 0.25$ and $p(B) = 0.75$, respectively), and between the SWOE for 4-shape and 1-shape estimates (Pearson correlation, $r = 0.70$ and 0.72, $N = 37$, $P < 10^{-10}$, for $p(B) = 0.25$ and $p(B) = 0.75$, respectively). Panel **c** shows a strong correlation between the SWOE extracted from choice behavior and 1-shape estimates (Pearson correlation, $r = 0.76$ and 0.83, $N = 37$, $P < 10^{-10}$, for $p(B) = 0.25$ and $p(B) = 0.75$, respectively), indicating that subjects used these shapes to make decisions consistent with their estimation. Panel **d** shows a correlation between the SWOE for 4-shape and 2-shape estimates (Pearson correlation, $r = 0.68$ and 0.72, $N = 37$, $P < 10^{-10}$, for $p(B) = 0.25$ and $p(B) = 0.75$, respectively). Panel **e** shows a correlation between the SWOE extracted from choice behavior and 2-shape estimates (Pearson correlation, $r = 0.70$ and 0.84, $N = 37$, $P < 10^{-10}$, for $p(B) = 0.25$ and $p(B) = 0.75$, respectively). Panel **f** shows a correlation between the SWOE extracted from choice behavior and 4-shape estimates (Pearson correlation, $r = 0.64$ and 0.68, $N = 37$, $P < 10^{-8}$, for $p(B) = 0.25$ and $p(B) = 0.75$, respectively).

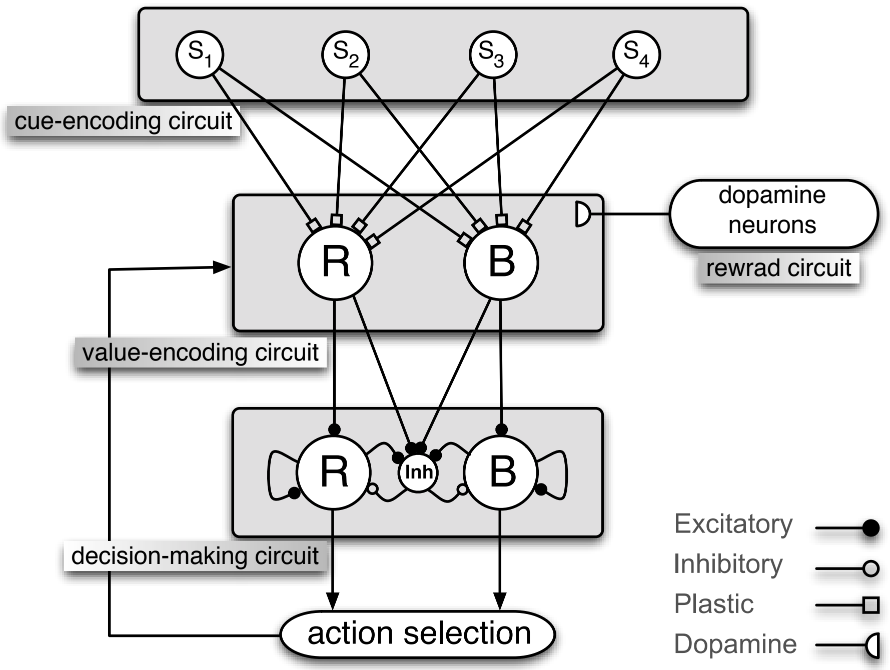

Supplementary Figure 5 | The schematic of our previous model of probabilistic inference. The model consists of four circuits: cue-encoding, value-encoding, decision-making, and reward. The cue-encoding circuit contains sensory neurons that are selective for individual visual cues (shapes), and project to the value-encoding circuit. The value-encoding circuit contains two pools of neurons that represent the reward value of the two alternative responses (action values) and project to the decision-making circuit. These neurons acquire such representation through their afferent plastic synapses that undergo reward-dependent Hebbian modifications (the same as in the current model but without the modulation by reward expectation). The decision-making circuit contains two competing neural pools that are selective for the two alternative responses (*B* and *R*, corresponding to blue and red targets, respectively), and an inhibitory pool of neurons. The reward circuit signals the presence or absence of reward at the end of each trial in a binary fashion.

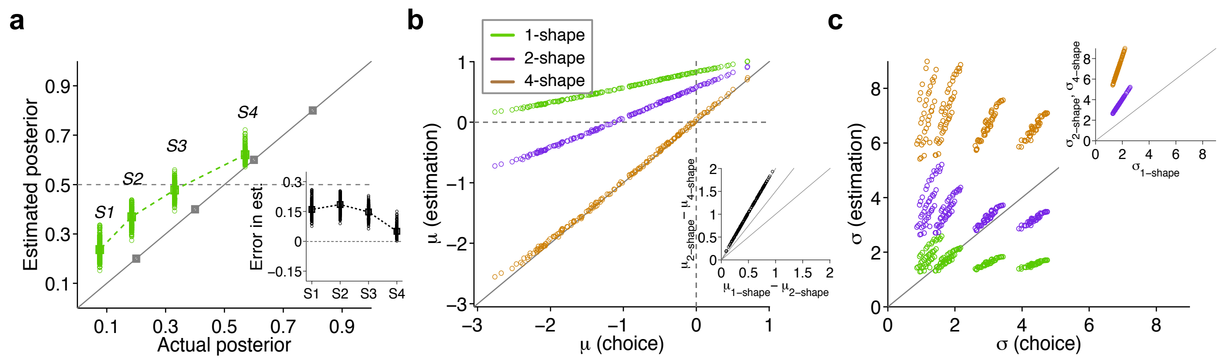

Supplementary Figure 6 | Behavior of the heuristic model over a wide range of parameters. Behavior of the heuristic model over a wide range of parameters (4 parameters, see Methods). Conventions are the same as in Figs. 5 and 6e-g. (**a**) The heuristic model's 1-shape estimates toward the less rewarding option as a function of the actual posteriors, over a wide range of parameters. (**b**) The heuristic model's estimation biases (relative to the more rewarding option) as a function of the bias in choice (relative to the more rewarding option). (**c**) The relationship between the heuristic model's stochasticity during estimation and choice. The heuristic model can perform the task and estimate posteriors associated with individual shapes with a bias toward the more rewarding option. However, the pattern of overestimation is different from experimental data. Moreover, due to the averaging mechanism, this model predicts that as the number of shapes used for estimation increases, the stochasticity in estimation should significantly increase (by a factor of 2 from 1-shape to 2-shape estimates and from 2-shape to 4-shape estimates).

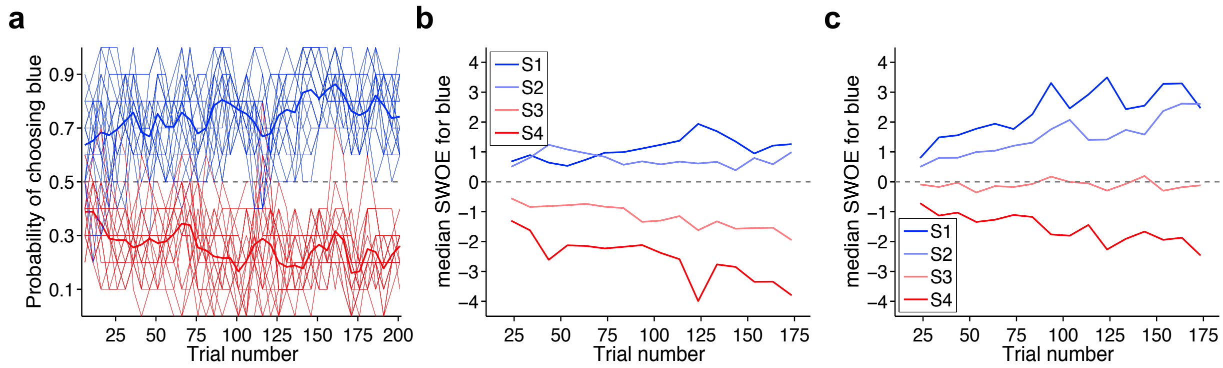

Supplementary Figure 7 | Learning about the two choice alternatives and predictive power of individual shapes over the course of the experiment. (**a**) The probability of choosing the blue target over time for individual subjects (thin curves) and average across all subjects (thick curves), separately for the two groups of subjects for whom the red or blue target was more rewarding. The probability of choosing blue for individual subjects was computed using a boxcar over 10 trials around a given trial. (**b-c**) Plotted are the medians of the SWOEs for individual shapes across two groups of subjects for whom the red (**b**) or blue (**c**) was more rewarding. The SWOEs were estimated from choice behavior of individual subjects over 45 trials around a given trial. More negative values in panel **b** and more positive values in panel **c** show a bias toward the more rewarding option in each case.

Supplementary Note 1

Control experiment with equal prior

In order to show that observed biases were due to the unequal prior probability of reward for the two choice alternatives, we also ran a control experiment in which 12 subjects performed the task with equal prior ($p(B) = 0.5$).

Firstly, we did not observe any significant biases during the choice and estimation sessions. More specifically, there was no significant biases in choice toward either option (two-sided signtest $P = 0.39$, $N = 12$), resulting in an unbiased average PF across subjects (Supplementary Fig. 2a). Similarly, there were no significant biases in estimation (two-sided signtest P = 1.0, 1.0, and 0.15 for 1-shape, 2-shape, and 4-shape estimates, respectively, $N = 12$). This is reflected in the distribution of μ values for individual subjects (Supplementary Fig. 3a-c) and in the average ePF over all subjects (Supplementary Fig. 2c).

Secondly, despite lack of any biases in choice or estimations, we looked for progression of biases as observed in the main experiment. We found that the biases for 1-shape estimates were not different from the biases for 2-shape estimates (two-sided signtest $P = 1.0$, $N = 12$). Moreover, the biases for 2-shape estimates were not different from the biases for 4-shape estimates (two-sided signtest $P = 0.39$, $N = 12$), and the biases in 4-shape estimates were similar to the biases in choice (two-sided signtest $P = 1.0$, $N = 12$).

Thirdly, we examined 1-shape estimates corresponding to the extent that individual shapes predicted the reward on a given option (Supplementary Fig. 2d). We found that subjects accurately predicted the posteriors for each shape and, except for S4 where there was a small underestimation (median = -0.05, two-sided signtest $P = 0.04$, $N = 12$), there was no significant difference between the estimated and actual values across subjects (two-sided signtest P = 0.23, 1.0, and 0.77 for S1, S2, and S3, respectively, $N = 12$). Note that this small difference is equal to half of the precision of the estimation report (estimations were reported in 10% steps). Similarly, the SWOE extracted from choice behavior (Supplementary Fig. 2b) were proportional to the actual log posterior odds, and except for S1, where there was a small overestimation (median = 0.1; two-

sided signtest $P = 0.04$, $N = 12$), there was no significant difference between the estimated and actual values across subjects (two-sided signtest $P = 0.51$, 0.51, and 0.50 for S2, S3, and S4, respectively, $N = 12$).

Finally, we quantified the stochasticity in choice behavior and estimation, measured by the σ values extracted from the PF of the choice session or the ePFs of the estimation session. We found a gradual increase in σ from 1-shape to 2-shape estimates (one-sided signtest $P = 0.02$, $N = 12$; $\sigma_{1\text{-}shape} = 0.79 \pm 0.46$, $\sigma_{2\text{-}shape} = 1.15 \pm 0.92$) and from 2-shape to 4-shape estimates (one-sided signtest $P = 0.07$, $N = 12$; $\sigma_{4\text{-}shape} = 1.47 \pm 0.77$; Supplementary Fig. 3d-f). The latter increase was not statistically significant at $P < .05$, probably due to a small sample size. Interestingly, the stochasticity in choice behavior and 4-shape estimates were statistically indistinguishable across all subjects (two-sided signtest $P = 0.39$, $N = 12$; $\sigma_{choice} = 1.06 \pm 0.65$).
